# The landscape of prognostic and immunological role of myosin light chain 9 (MYL9) in human tumors

**DOI:** 10.1002/iid3.557

**Published:** 2021-11-02

**Authors:** Minghe Lv, Lumeng Luo, Xue Chen

**Affiliations:** ^1^ Department of Radiation Oncology Fudan University Shanghai Cancer Center, Fudan University Shanghai China; ^2^ Department of Oncology, Shanghai Medical College Fudan University Shanghai China

**Keywords:** cancer, immune infiltration, MYL9, prognosis

## Abstract

**Introduction:**

Recent studies have shown that myosin light chain 9 (MYL9) plays a vital role in immune infiltration, tumor invasion, and metastasis; however, the prognostic and immunological role of MYL9 has not been reported. The purpose of this study was to explore the potential prognostic and immunological roles of MYL9 in human cancers by public datasets mainly including the cancer genome atlas (TCGA) and Gene expression omnibus.

**Methods:**

The expression pattern and prognostic value of MYL9 were analyzed across multiple public datasets in different cancer. The correlations between MYL9 expression and immune infiltration among multiple cancers were analyzed by using the TIMER2.0. The MYL9‐related gene enrichment analysis was implemented by mainly using KEGG and GO datasets.

**Results:**

MYL9 was lowly expressed in most cancers, such as breast cancer, lung adenocarcinoma and squamous cell carcinoma, and stomach adenocarcinoma; but it was highly expressed in several cancers, such as cholangiocarcinoma, head and neck squamous cell carcinoma, and liver hepatocellular carcinoma. Furthermore, MYL9 expression was distinctively associated with prognosis in adrenocortical carcinoma, colon adenocarcinoma, brain glioma, lung cancer, ovarian cancer, gastric cancer, breast cancer, blood cancer, and prostate cancer patients. The expressions of MYL9 were significantly associated with the infiltration of cancer‐associated fibroblasts, B cell, CD8^+^T cell, CD4^+^T cell, macrophage, neutrophil, dendritic cell in different tumors as well as immune markers. In addition, we found that the functional mechanisms of MYL9 involved muscle contraction and focal adhesion.

**Conclusion:**

MYL9 can serve as a prognostic signature in pan‐cancer and is associated with immune infiltration. This pan‐cancer study is the first to show a relatively comprehensive understanding of the prognostic and immunological roles of MYL9 across different cancers.

## Graphical abstract

Our first pan‐cancer analyses of MYL9 indicated statistical correlations of MYL9 expression with clinical prognosis, immune cell infiltration across multiple tumors, which aids in understanding the role of MYL9 in tumorigenesis from the perspective of clinical tumor samples. Our results indicated that MYL9 can serve as a prognostic signature in pan‐cancer and is associated with immune infiltration.

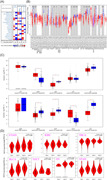

## 1 INTRODUCTION

Due to the complexity of tumor genesis and development, it is very important to analyze the expression of any valuable genes and evaluate their correlation with clinical survival prognosis and possible molecular mechanisms. The public databases, such as TCGA^1^, ^2^ and Gene expression omnibus (GEO), contain functional genomics datasets for different cancers,^3^, ^4^ and that set the stage for us to do a generalized cancer analysis.

Myosin is an actin‐dependent molecular motor that uses the energy of adenosine triphosphatase hydrolysis to move along actin filaments and generate force. Myosin has many functions, including cell signaling, cell contractility, vesicle trafficking, endocytosis, and protein/RNA localization.^5^, ^6^ Myosin consists of two heavy chains and four light chains. Myosin light chain 9 (MYL9), a regulatory subunit of the forcing‐producing ATPase nonmyosin II (NMII),^7^ may regulate muscle contraction by regulating ATPase activity in the myosin head. It binds to actin filaments to control cytoskeletal dynamics and is subsequently involved in cell shape establishment, migration, polarity, adhesion, and signal‐mechanical transduction.^8^, ^9^ Recently, many studies showed that MYL9 played the role of a promoter in tumor invasion and metastasis.^10^ For example, the MAL/SRF complex was involved in platelet formation and megakaryocyte migration by regulating MYL9 (MLC2) and MMP9,^11^ suggesting that it has clinical significance in human tissues of different tumor types. In addition, phosphorylation of MYL9 is known to be key to cell migration on solid substrates.^12^ Therefore, MYL9 may play a crucial role in the genesis and development of tumors. However, based on large clinical data, there is currently no evidence of a generalized relationship between MYL9 and multiple tumor types.

In this study, we attempted to mainly use the TCGA and GEO databases to explore the prognostic and immunological role of MYL9 across different tumors, and to investigate the potential molecular mechanism of MYL9 in the pathogenesis or clinical prognosis of different tumors via gene expression, survival prognosis, immune infiltration, and enrichment analysis.

## 2 METHODS

### 2.1 The analysis of MYL9 gene expression

We first used the Oncomine dataset to obtain the expression of MYL9 between cancers and corresponding normal tissues (https://www.oncomine.org/). Then, the TIMER2.0 (tumor immune estimation resource; version 2) web (http://timer.cistrome.org/) and GEPIA2 (Gene Expression Profiling Interactive Analysis; version 2) web server (http://gepia2.cancer-pku.cn/#analysis) were used to further analyze the expression difference between human tumors and normal tissues. In GEPIA2, we set *p*‐value cutoff = 0.01, log2FC (fold change) cutoff = 1, and “Match TCGA normal and GTEx data.” Furthermore, we also employed the GEPIA2 to observe the correlation between MYL9 expression and the pathological stages (Stage I, Stage II, Stage III, and Stage IV) of cancers, and the results were shown by the box or violin plots.

### 2.2 The survival prognosis analysis of MYL9

The prognostic role of MYL9 was firstly analyzed by the “Survival Map” module of GEPIA2. Then, we used the data from Prognoscan (http://www.abren.net/PrognoScan/) and Kaplan–Meier plotter (https://kmplot.com/analysis/) databases to further analyze the effects of MYL9 expression on prognosis in different tumors. Kaplan–Meier plotter tool was used to analyze the effects of clinicopathological factors and MYL9 on the prognosis of patients with gastric cancer and ovarian cancer.

### 2.3 Immune infiltrating analysis and prognosis analysis

The association between MYL9 expression and cancer‐associated fibroblasts (CAFs) across all TCGA tumors was obtained by using the TIMER2 tool. The EPIC, MCPCOUNTER, XCELL, and TIDE algorithms were used to estimate immune penetration. The *p* and partial correlation (COR) values were obtained by the Spearman rank correlation test with purity adjustment. The data were visualized as a heatmap and a scatter plot. Furthermore, we employed the TIMER2 tool to set a Cox Proportional Hazard Model to evaluate CAF, age, stage, gender, race, purity, and MYL9, via the EPIC, MCPCOUNTER, XCELL, and TIDE algorithms. Additionally, the relationship between MYL9 expression and immune infiltrating cells, including B cell, CD8^+^T cell, CD4^+^T cell, macrophage, neutrophil, dendritic cell (DC) were determined by using the TIMER (http://cistrome.org/TIMER/) databases. We further obtained the Kaplan–Meier curve for cumulative survival (CS), under the settings of 50% split expression percentage of patients, 50% split infiltration percentage of patients, and survival time between 0 and 200 months.

### 2.4 The enrichment analysis of MYL9‐related gene

We first used the STRING website (https://string-db.org/) to obtain the Top 50 of MYL9 binding proteins. Then, using the GEPIA2 website, we obtained the top 100 of MYL9‐related targeted genes based on the data set of all TCGA cancers. We further used the GEPIA2 tool to conduct a pairwise gene Pearson correlation analysis of MYL9 and selected genes. Moreover, we used the TIMER2 tool to offer the heatmap data of these genes, which contains the partial correlation (cor) and *p* value in the purity‐adjusted Spearman's rank correlation test. We use bioinformatics and evolutionary genomics website (http://bioinformatics.psb.ugent.be/webtools/Venn/) interaction analysis to compare MYL9 combination and interaction of genes. Moreover, KEGG (Kyoto Encyclopedia of Genes and Genomes) path analysis and GO (Gene Ontology) analysis were performed based on the two sets of data. The gene lists were uploaded to DAVID (database for annotation, visualization, and integrated discovery) for the data of the functional annotation chart. The bubble plots of enriched pathways were finally visualized with the bioinformatics web (http://www.bioinformatics.com.cn/).

### 2.5 Statistical analysis

Data from the Oncomine database was presented as *p* values determined by *t* test, fold changes, and Gene Rank. We used the PrognoScan, Kaplan–Meier plotter, TIMER and TIMER2, and GEPIA2 websites to conduct survival figures in respective analyses, with data including either hazard ratio (HR) and *p* values or *p* values derived from a log‐rank test. Spearman's and Pearson correlation analyses were used to gauge the degree of correlation between particular variables. MYL9‐related gene enrichment analysis was analyzed by using KEGG and GO databases. *p* < .05 was considered statistically significant, if not specially noted.

## 3 RESULTS

### 3.1 The analysis of MYL9 gene expression in different cancers

**Figure 1:**
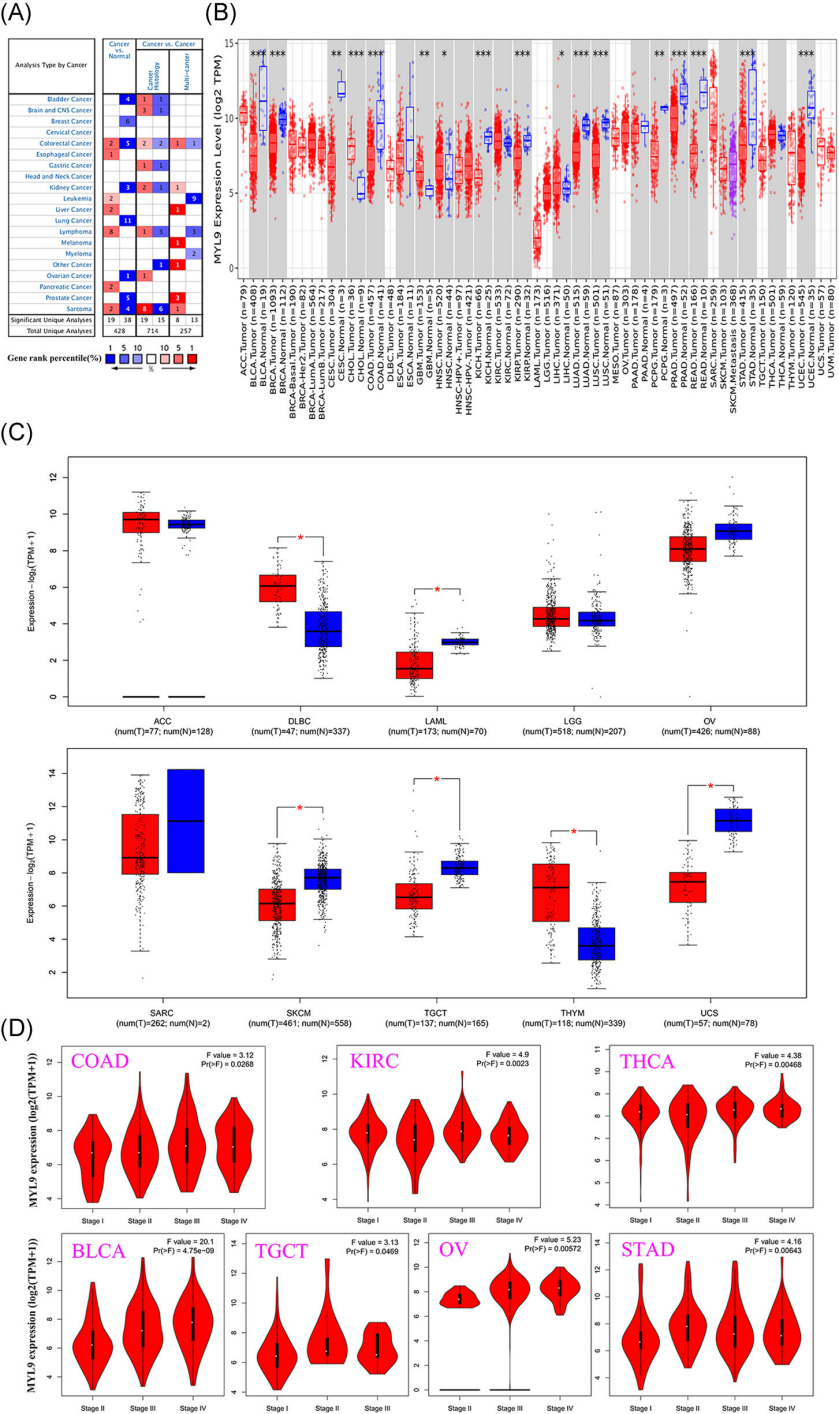
The expression level of myosin light chain 9 (MYL9) in different tumors. (A) The expression level of MYL9 in different types of cancer and normal tissues by using the Oncomine database (the threshold of the *p* value is .0001, fold change is 2, and gene ranking is top 10%). (B) The expression of the MYL9 gene was analyzed by TMIER 2.0 in different cancers. (C) The box plot of MYL9 gene expression was supplied for different tumors by using the TCGA and GTEx database. (D) The expression levels of the MYL9 gene were analyzed by the main pathological stages of colon adenocarcinoma (COAD), kidney renal clear cell carcinoma (KIRC), thyroid carcinoma (THCA), bladder urothelial carcinoma (BLCA), testicular germ cell tumors (TGCT), ovarian serous cystadenocarcinoma (OV), and stomach adenocarcinoma (STAD). Log2 (TPM + 1) was applied for log‐scale. * *p* < .05; ** *p* < .01; *** *p* < .001, **** *p* < .0001

In this study, we first assessed the expression of MTL9 in multiple tumors and normal tissue types using the Oncomine database, and results revealed that the expression of this gene was reduced compared with normal tissues for bladder, breast, kidney, lung, ovarian, and prostate cancers, but was elevated compared with normal tissues for esophageal cancer, leukemia, liver cancer, lymphoma, and pancreatic cancer (Figure 1A). We additionally used the TIMER2.0 tool to analyze the expressions of the MTL9 gene in TCGA data set for all types of cancers, and found that the expressions of MYL9 gene in BLCA (bladder urothelial carcinoma), BRCA (breast invasive carcinoma), CESC (cervical squamous cell carcinoma and endocervical adenocarcinoma), COAD (colon adenocarcinoma), KICH (kidney chromophobe), KIRP (kidney renal papillary cell carcinoma), LUAD (lung adenocarcinoma), LUSC (lung squamous cell carcinoma), PCPG (pheochromocytoma and paraganglioma), PRAD (prostate adenocarcinoma), READ (rectum adenocarcinoma), STAD (stomach adenocarcinoma), UCEC (uterine corpus endometrial carcinoma) were significantly lower than the corresponding control tissues; however, the expressions of MYL9 gene in CHOL (cholangiocarcinoma), GBM (glioblastoma multiforme), HNSC (head and neck squamous cell carcinoma), LIHC (liver hepatocellular carcinoma) were obviously elevated (Figure 1B). Due to the lack of the normal tissue of ACC (adrenocortical carcinoma), DLBC (lymphoid neoplasm diffuse large B‐cell lymphoma), LAMC (acute myeloid leukemia), LGG (brain lower grade glioma), OV (ovarian serous cystadenocarcinoma), SARC (sarcoma), SKCM (skin cutaneous melanoma), TGCT (testicular germ cell tumors), THYM (thymoma), and UCS (uterine carcinosarcoma) in the TCGA database, we further evaluated the difference in MYL9 expression between normal and tumor tissues among ACC, DLBC, LAMC, LGG, OV, SARC, SKCM, TGCT, THYM, and USC, by using normal tissues from the GTEx data set as controls, and results showed that the expressions of MTL9 gene in DLBC and THYM were higher than corresponding normal tissues, but the expressions of MTL9 gene in LAMC, SKCM, TGCT, and UCS were lower than normal control tissues (Figure 1C). We also used the "pathological staging map" module of GEPIA2 to observe the correlation between MYL9 expression and tumor pathological staging, including COAD, KIRC (kidney renal clear cell carcinoma), THCA (thyroid carcinoma), BLCA, TGCT, OV, STAD (Figure 1D). The data of this part indicated that the MYL9 gene played a different role in different tumors, which deserved further investigation.

### 3.2 Survival analysis data of MYL9 gene in different cancers

**Figure 2:**
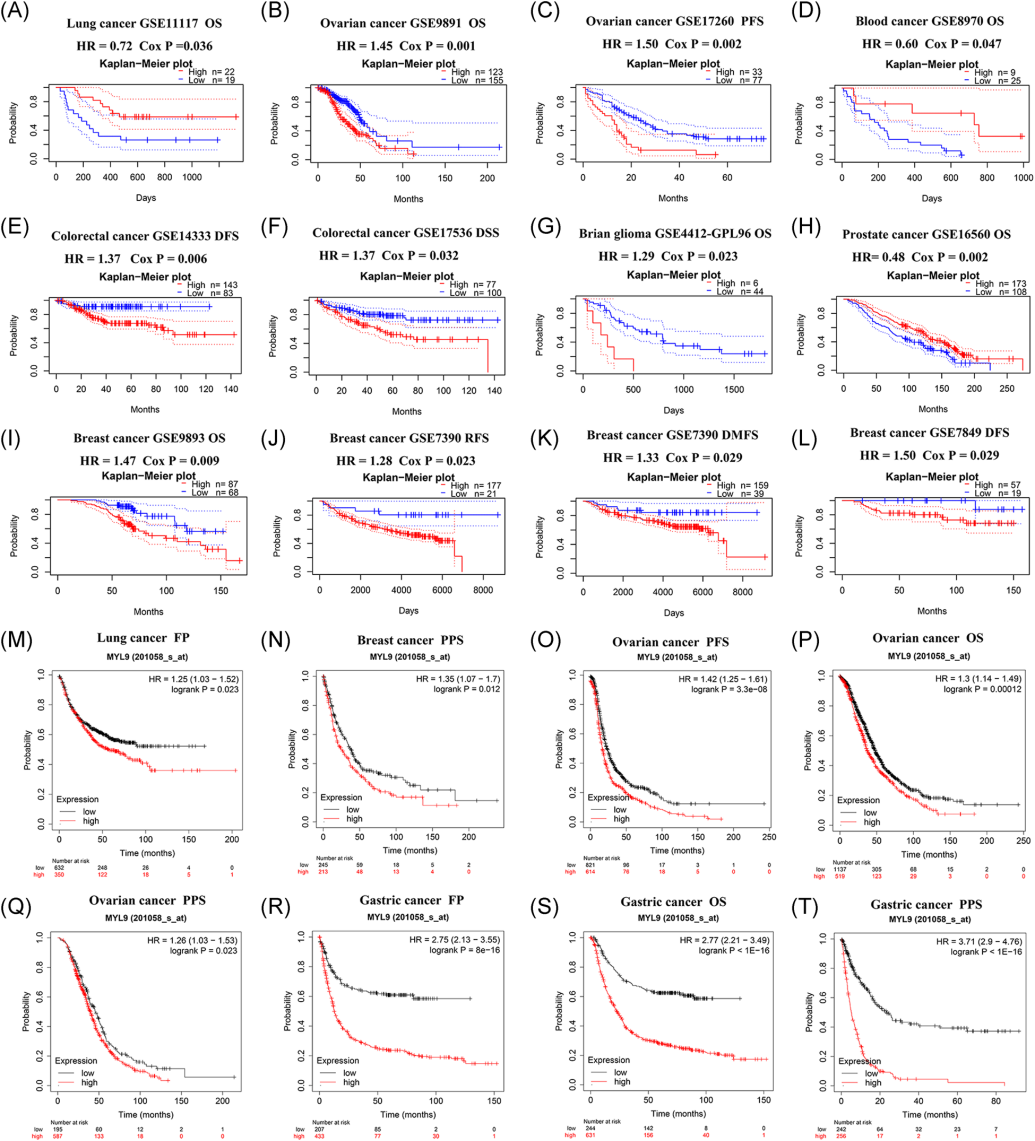
The prognosis analysis of myosin light chain 9 (MYL9) gene expression in PrognoScan and Kaplan–Meier plotter datasets for different tumors. The effects of MYL9 expression on prognosis among various types of cancers in the PrognoScan dataset (A–L) and Kaplan–Meier database (M–T). DFS, disease‐free survival; DMFS, distant metastasis‐free survival; DSS, disease‐specific survival; FP, first progression; OS, overall survival; PFS: progression‐free survival; PPS: postprogression survival; RFS, relapse‐free survival

To further explore the effects of the MTL9 gene on different tumors, we divided tumor cases into high‐expression group and low‐expression group according to the expression level of MYL9 and used the GEPIA2 tool to investigate the effects of the MYL9 gene on overall survival (OS) and disease‐free survival (DFS). The heatmap and Kaplan–Meier plot of OS in different tumors were displayed, and results showed that the low MYL9 group had a longer OS than the high MYL9 group in COAD, LGG, and MESO (mesothelioma) patients; however, the results were adverse in ACC patients. We further found that lowly expressed MYL9 was linked to favorable prognosis of DFS for COAD patients, but seemingly suggested a poor prognosis of DFS for THYM patients (Figure S1). To further investigate and verify the effects of MYL9 on prognosis in different tumors, we obtained the survival analysis data of MYL9 in PrognoScan (Figure S2–6) and Kaplan–Meier datasets. We found that in the Prognoscan data set, multiple cancers showed a marked correlation between the prognosis of patients and the expression level of MYL9 including lung, ovarian, blood, prostate, brain, breast, and colorectal cancer (Figure 2A–L). We also used the Kaplan–Meier plotter dataset to assess how the expression of MYL9 related to prognosis in a series of tumor types, revealing that its reduction was significantly linked with a greater OS in ovarian cancer and gastric cancer, a greater first progression (FP) in lung cancer and gastric cancer, and a better post progression survival (PPS) in breast cancer, ovarian cancer and gastric cancer (Figure 2M–T). In addition, we used the data of Kaplan–Meier plotter RNA‐seq dataset to further study the effects of the mRNA expression levels of MYL9 on prognosis in different tumors, results showing that its reduction was obviously linked with a better OS and relapse‐free survival (RFS) in the majority of cancers except for sarcoma (Figure S7). Therefore, our pan‐cancer analysis showed that MYL9 is a potential prognostic factor that contributed to the clinical treatment and prevention of tumors.

### 3.3 The relationship between MYL9 expression and patient clinicopathological findings in Kaplan–Meier plotter dataset

In previous results, we found that the low‐expression level of MYL9 was linked to the great prognosis of gastric and ovarian cancer patients in the Kaplan–Meier plotter dataset. Therefore, we attempted to explore the underlying mechanisms by using the Kaplan–Meier plotter database and to assess the relationship between the expression of MYL9 and the clinicopathological factors of cancer patients. As shown in Table 1, we found that the expression level of MYL9 correlated markedly with OS and PPS, and with sex, TNM stage, Lauren classification, and HER2 status in gastric cancer except for mixed Lauren classification. We further found that the expression level of MYL9 related to each *N* stage, corresponding to the degree of lymph node metastasis in gastric cancer patients. This lymph node metastasis was the most common type of metastasis in gastric cancer patients and was directly related to the prognosis of patients. As for the relationship between MYL9 and PPS in gastric cancer, HR in Stage N was the highest, indicating that MYL9 expression may affect the prognosis of gastric cancer patients by affecting lymph node metastasis. As shown in Table 2, we found that the expression of MYL9 related markedly to OS and PFS and to stage and histology in ovarian cancer. In particular, as for the relationship between MYL9 and OS in ovarian cancer, histology exhibited the highest HR, and for the relationship between MYL9 and PFS in ovarian cancer, Stage I showed the highest HR.

**Table 1:** The effects of different clinicopathological factors on MYL9 gene expression and clinical prognosis were determined by Kaplan–Meier plotter

Clinicopathological characteristics	Overall survival (*n* = 875)			postprogression survival (*n* = 498)		
	*N*	Hazard ratio	*p* value	*N*	Hazard ratio	*p* value
Sex						
Male	544	2.96 (2.25–3.9)	6.60E–16	348	3.83 (2.86–5.13)	1.00E–16
Female	236	2.65 (1.77–3.97)	8.30E–07	149	3.5 (2.22–5.53)	1.30E–08
Stage						
I	67	3.99 (1.27–12.56)	.011	31	‐	‐
II	140	2.3 (1.26–4.19)	.0052	105	2.75 (1.42–5.33)	.0018
III	305	2.7 (1.82–3.99)	2.50E–07	142	4.2 (2.54–6.95)	1.90E–09
IV	148	1.87 (1.21–2.9)	.0043	104	2 (1.26–3.18)	.0028
Stage T						
2	241	2.11 (1.37– 3.23)	.00046	196	3.01 (1.92–4.72)	4.70E–07
3	204	2.06 (1.42–3)	.00011	150	2.62 (1.72–3.98)	3.00E–06
4	38	3.48 (1.36−8.93)	.0061	29	1.89 (0.67–5.32)	.22
Stage N						
0	74	3.09 (1.19–7.98)	.015	41	7.85 (2.09–29.43)	.00034
1	225	3.45 (2.22–5.35)	4.10E–09	169	5.25 (3.23–8.55)	1.60E–13
2	121	3.1 (1.91–5.04)	1.70E–06	105	3.26 (1.94–5.47)	2.70E–06
3	76	2.15 (1.23–3.78)	.0064	63	2.27 (1.24–4.15)	.0061
1 + 2 + 3	422	2.73 (2.06–3.63)	5.00E–13	337	3.37 (2.5–4.54)	1.00E–16
Stage M						
0	444	2.5 (1.87–3.33)	1.00E–10	342	3.81 (2.78–5.22)	1.00E–16
1	56	2.21 (1.2–4.07)	.0095	36	3.38 (1.48–7.68)	.0024
Lauren classification						
Intestinal	320	3.03 (2.14–4.28)	5.10E–11	192	4.49 (2.94–6.84)	2.80E–14
Diffuse	241	2.39 (1.67–3.41)	8.70E–07	176	2.69 (1.8–4.03)	5.10E–07
Mixed	32	3.79 (1.2–11.97)	1.50E–02	16	‐	‐
Differentiation						
Poor	165	1.63 (0.95–2.79)	.071	49	1.5 (0.73–3.06)	.27
Moderate	67	1.77 (0.92–3.39)	.081	24	0.62 (0.22– 1.78)	.37
Well	32	6.46 (2.13−19.56)	.00018	0	‐	‐
HER2 status						
HER2 negative	532	2.59 (2–3.35)	6.60E–14	334	3.38 (2.5– 4.57)	1.00E–16
HER2 positive	343	1.81 (1.3–2.52)	4.00E–04	164	4 (2.51–6.36)	3.90E–10

Abbreviation: MYL9, myosin light chain 9.

**Table 2:** The effects of different clinicopathological factors on MYL9 gene expression and clinical prognosis of ovarian cancer were determined by Kaplan–Meier plotter

Clinicopathological characteristics	Overall survival (*n* = 1656)			Progression‐free survival (*n* = 1435)		
	*N*	Hazard ratio	*p* value	*N*	Hazard ratio	*p* value
Stage						
I	74	2.54 (0.68−9.45)	.15	96	3.91 (1.36−11.24)	.006
I + II	135	1.99 (0.75−5.29)	.16	163	1.84 (1.01−3.33)	.042
II	61	3.38 (0.73−15.57)	.1	67	1.46 (0.65−3.25)	.36
II + III	1105	1.29 (1.09−1.53)	.003	986	1.49 (1.28−1.73)	1.7E−07
II + III + IV	1281	1.34 (1.12−1.59)	.001	1148	1.54 (1.34−1.77)	7.8E−10
III	1044	1.27 (1.07−1.51)	.006	919	1.49 (1.28−1.74)	3.7E−07
III + IV	1220	1.26 (1.07−1.47)	.004	1081	1.54 (1.34−1.77)	2E−09
IV	176	2.81 (1−7.86)	.042	162	1.88 (1.29−2.74)	.001
Histology						
Endometrioid	37	7.65 (1.26−46.43)	.009	51	3.75 (1.45−9.7)	.004
Serous	1207	1.29 (1.1−1.51)	.001	1104	1.61 (1.39−1.86)	9.3E−11
TP53 mutation						
Mutated	509	1.35 (1.03−1.76)	.026	483	1.43 (1.14−1.78)	.002
Wild type	94	1.61 (0.94−2.77)	.08	84	1.75 (1.02−3.02)	.04
Debulk						
Optimal	801	1.52 (1.23−1.88)	9.7e−05	696	1.49 (1.23−1.8)	4.3E−05
Suboptimal	536	1.21 (0.96−1.51)	.099	459	1.25 (1−1.57)	.046

Abbreviation: MYL9, myosin light chain 9.

### 3.4 Correlation analysis between the expression of MYL9 and immune cell infiltrating

**Figure 3:**
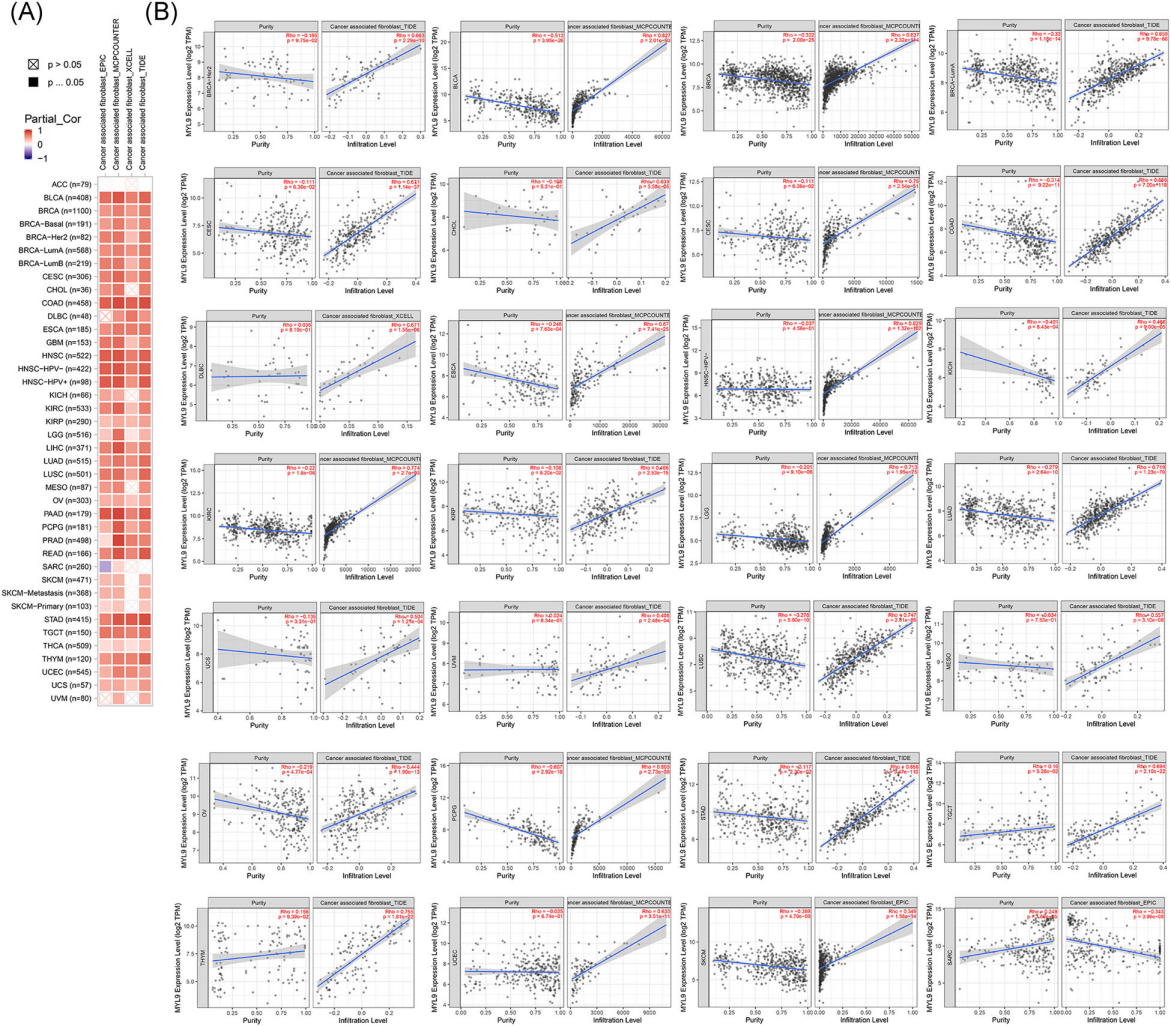
The expression level of myosin light chain 9 (MYL9) correlated with the immune infiltrating level of cancer‐associated fibroblasts. Through the EPIC, MCPCOUNTER, XCELL, and TIDE algorithms, the heatmap about the potential relationship between the infiltration level of different immune cells and MYL9 gene expression was exhibited by using the TIMER2.0 tool in different tumors. The heatmap (A), and the scatter plot (B) with the highest Spearman's *p* value results in four datasets were given

Many studies showed that the occurrence and development of tumors were related to tumor‐infiltrating immune cells in the immune microenvironment. Therefore, in this part, we further explored the relation of MYL9 expression among immune infiltration of CAFs. Through the EPIC, MCPCOUNTER, XCELL, and TIDE algorithms, the heatmap about the potential relationship between the infiltration level of CAFs and MYL9 gene expression was exhibited by using the TIMER2.0 tool in different tumors (Figure 3A). As shown in Figure 3B, the correlation between CAFs and MYL9 expression was shown by using a scatter diagram. We found that only in SARC, the expressions of MYL9 was negatively related with the infiltration level of CAFs based on the TIDE algorithm, while the expressions of MYL9 gene in other tumors were positively correlated with the expressions of CAFs, including BRCA‐Her2 with the TIDE algorithm, BLCA with the MCPCOUNTER algorithm, BRCA‐LumA with the TIDE, CESC with the TIDE algorithm, CHOL with the TIDE algorithm, COAD with TIDE algorithm, DLBC with the XCELL algorithm, ESCA with the MCPCOUNTER algorithm, HNSC‐HPV^+^ with the MCPCOUNTER algorithm, KICH with the TIDE algorithm, KIRC with the MCPCOUNTER algorithm, KIRP with the TIDE algorithm, LGG with the MCPCOUNTER algorithm, LUAD with TIDE algorithm, UCS with the TIDE algorithm, UVM with the TIDE algorithm, LUSC with the TIDE algorithm, MESO with the TIDE algorithm, OV with the TIDE algorithm, PCPG with the MCPCOUNTER algorithm, STAD with the TIDE algorithm, TGCT with the TIDE algorithm, THYM with the TIDE algorithm, UCEC with the MCPCOUNTER algorithm, SKCM with the EPIC algorithm. To further investigate the correlation between MYL9 expression and immune infiltrating, we employed the TIMER tool to analyze the correlation of B cell, CD8^+^T cell, CD4^+^T cell, macrophage, neutrophil, DC with MYL9 expression, and results showed that the expression of MYL9 significantly correlated with the infiltration of macrophage in BLCA, with the infiltration of CD4^+^T cell and macrophage, neutrophil, DC in COAD, with the infiltration of macrophage in ESCA, with the infiltration of DC in GBM, with the infiltration of macrophage in HNSC, with the infiltration of CD4^+^T cell and macrophage in HNSS‐HPV^−^, with the infiltration of macrophage in KIRP, with the infiltration of CD4^+^T cell, with the infiltration of CD4^+^T cell and macrophage in LIHC, with the infiltration of macrophage, neutrophil, and DC in LUAD, with the infiltration of CD4^+^T cell, macrophage, neutrophil, and DC in LUSC, with the infiltration of macrophage, neutrophil, and DC in PAAD, with the infiltration of CD4^+^T cell and macrophage in READ, and, with the infiltration of CD4^+^T cell, macrophage, and DC in STAD (Figure S8). All data of this part indicated that MYL9 expression correlated with immune infiltration in different tumors, which might be a potential mechanism to exert the effects on prognosis.

### 3.5 Correlations between the expression of MYL9 and immune cell markers

**Figure 4:**
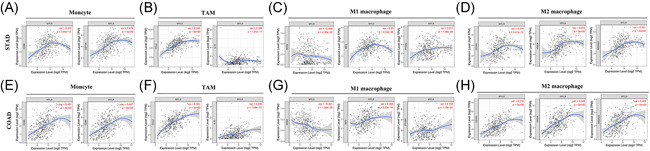
The expression of myosin light chain 9 (MYL9) was correlated with the immunological markers in gastric adenocarcinoma (STAD) and colon adenocarcinoma (COAD). (A–D) Scatter diagram of relations between the expression of MYL9 and the markers of monocytes (A), TAMs (B), M1 macrophage (C), and M2 macrophages (D) in STAD. (E–H) Scatter diagram of relations between the expression level of MYL9 and the markers of monocytes (E), TAM (F), M1 macrophage (G), and M2 macrophages (H) in COAD

To further investigate the potential relation between the expression level of MYL9 and infiltrating immune cells, we explored the relationships between the expression of MYL9 and the markers of several immune cells by using the GEPIA and TIMER tools. These markers were used to characterize immune cells, including CD8^+^ T and CD4^+^ T cell, B cell, M1/M2 macrophage, monocyte, neutrophil, NK, tumor‐associated macrophage, and DC in STAD and COAD. We also analyzed the different functional T cells such as Treg, Th1, Th2, Th9, Th17, Th22, Tfh, and exhausted T cells. We found that in GAPIA, the MYL9 expression level obviously related to 61 out of 77 immune cell markers in STAD, and related to 47 out of 77 immune cell markers in the corresponding normal tissues. In COAD, the expression levels of MYL9 obviously correlated with 68 out of 77 immune cell markers and correlated with 23 out of 77 immune cell markers in corresponding normal tissues (Table S1). Elevated MYL9 expression was associated with increased DC infiltration in STAD and COAD, and consistent with this, the DC markers including CD1C, CD141, HLA‐DPB1, HLA‐DRA1, BDCA‐4(NRP1), and CD11c(ITGAX) linked with the expression level of MYL9. This suggested that the expression level of MYL9 was closely related to the penetration of tumor DC. We additionally observed that there was a marked relation between the expression of MYL9 and the markers of Tregs and exhausted T cells including FOXP3, CCR8, CD25 (IL2RA), STAT5B, TGFβ (TGFB1), PD1 (PDCD1), CTLA4, LAG3, TIM‐3(HAVCR2), GZMB in STAD and COAD (Table S1), indicating that MYL9 had a potential role in immune escape in STAD and COAD, although further research would be needed to demonstrate the mechanisms underlying such escape. Interestingly, MYL9 expression markedly correlated with TAM and M2 macrophage markers in STAD as well as the corresponding normal tissues; however, MYL9 expression significantly correlated with monocyte marker in STAD but the two were irrelevant in corresponding normal tissues. In COAD, we found in particular that MYL9 expression significantly correlated with the M2 macrophage marker, but the two were irrelevant in corresponding normal tissues. So, we used the TIMER tool to further analyze the relation between MYL9 expression and monocyte, TAM, M1 macrophage, and M2 macrophage markers, and this result brought into correspondence with the results in GAPIA. As shown in Figure 4, the expression level of MYL9 obviously related to monocyte markers (CD86, CD115), TAM markers (CCL2, IL10), M1 macrophage marker (IRF5), and M2 macrophage markers (CD163, VSIG4, MS4A4A) in STAD (Figure 4A–D) and COAD (Figure 4E–H). Therefore, we speculate that MYL9 may have the function of regulating the polarization of macrophages.

### 3.6 The prognosis analysis of MYL9 gene expression and immune infiltration cells in different tumors

**Figure 5:**
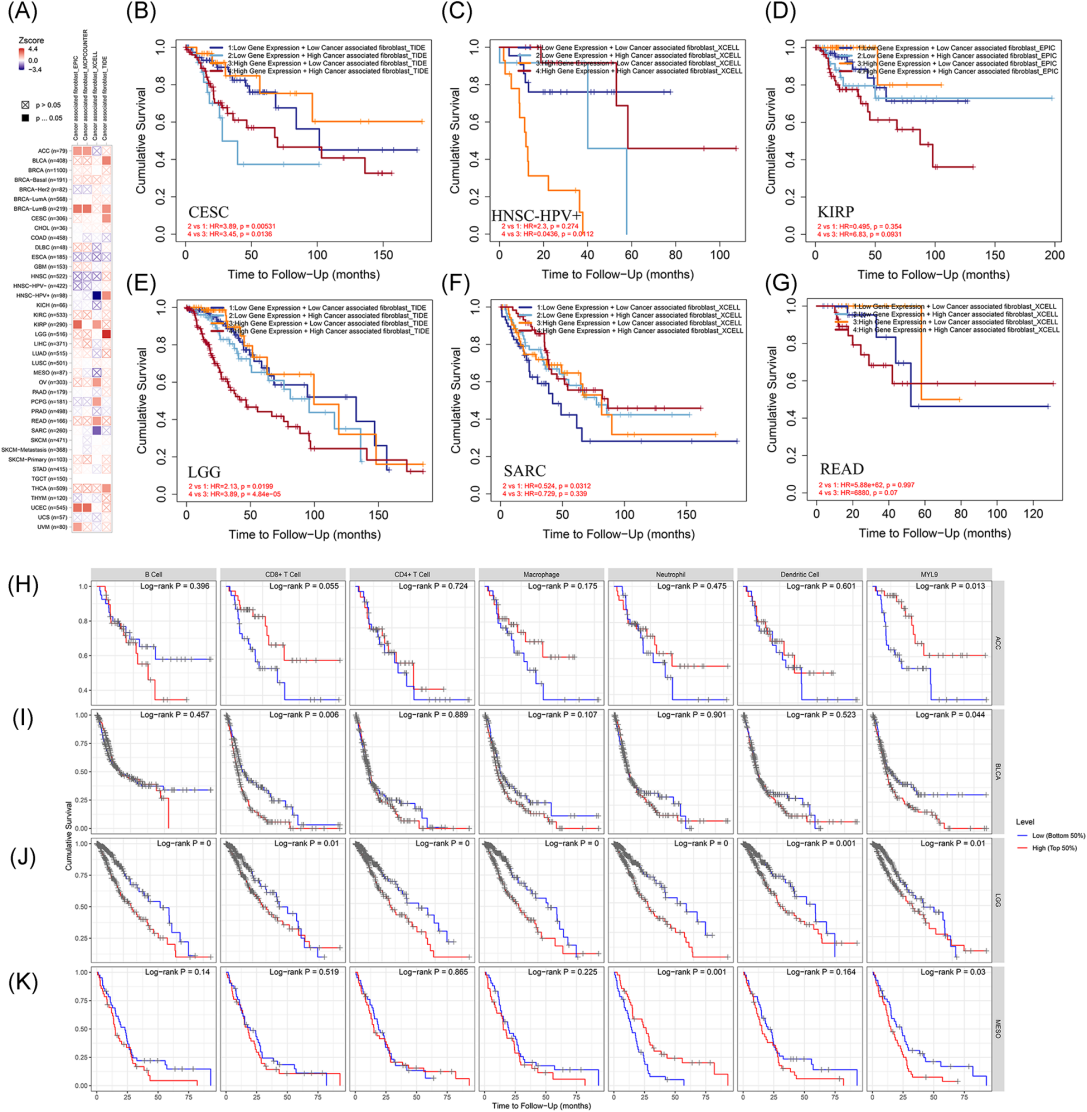
The prognosis analysis of myosin light chain 9 (MYL9) gene expression and immune infiltration cells in different tumors. (A) The heatmap about multivariable Cox model analysis of MYL9, cancer‐associated fibroblasts, age, stage, gender, race, and purity for outcome in different tumors. The Kaplan–Meier curve of MYL9 expression and cancer‐associated fibroblast immune infiltrating in cervical squamous cell carcinoma and endocervical adenocarcinoma (CESC) (B), HNS‐HPV^+^(C), kidney renal papillary cell carcinoma (KIRP) (D), brain lower grade glioma (LGG) (E), sarcoma (SARC) (F), and READ (G) via using the TIMER2.0 tool. The effects of the immune infiltrating of B cell, CD8^+^T cell, CD4^+^T cell, macrophage, neutrophil, dendritic cell, and the expression of MYL9 on cumulative survival in adrenocortical carcinoma (ACC) (H), bladder urothelial carcinoma (BLCA) (I), LGG (J), and mesothelioma (MESO) (K)

In previous results, we elaborated that the expression levels of the MYL9 gene were significantly correlated with the immune infiltrating in CAF. In this part, we attempted to explore the prognosis effects of MYL9 and CAF in the TCGA data set of different tumors. As shown in Figure 5A, a heatmap presented the normalized coefficient of the infiltrate for each Cox proportional hazard model across multiple cancer types. To move forward a single step, we found that in CESC, the CS of the low MYL9 expression and low CAF group was markedly longer than the low MYL9 expression and high CAF group; however, the CS of the high MYL9 expression and high CAF group was obviously shorter than the high MYL9 expression and low CAF group (Figure 5B). As shown in Figure 5C, interestingly, the CS of the high MYL9 expression and high CAF group was longer than the high MYL9 expression and low CAF group in HNSS‐HPV^+^, but there was no significant difference between groups of KIRP (Figure 5D). In LGG, there was longer CS in the low MYL9 expression and low CAF group than the low MYL9 expression and high CAF group, and, the CS of the high MYL9 expression and low CAF fibroblast group was longer than the high MYL9 expression and high CAF group (Figure 5E). As shown in Figure 5F, we found that the CS of the low MYL9 expression and low CAF group was significantly longer than the low MYL9 expression and high CAF group in SARC, but there was no significant difference between groups of READ (Figure 5G). The data of this part indicated that MYL9 expression and CAF played an important role in CS of cancer patients, and their effects were different or adverse on different types of tumors, which would provide potential and novel targeting for clinical cancer diagnosis and therapy, expecting to improve the prognosis of cancer patients.

We further studied the effects of the immune infiltrating of B cell, CD8^+^T cell, CD4^+^T cell, Macrophage, Neutrophil, DC, and the expression of MYL9 on CS in different cancers (Figure S9), and results showed that MYL9 expression was significantly correlated with prognosis in ACC (Figure 5H), CD8^+^T cell infiltration and MYL9 expression were obviously correlated with prognosis in BLCA (Figure 5I), B cell, CD8^+^T cell, CD4^+^T cell, Macrophage, Neutrophil, DC, and the expression of MYL9 were significantly correlated with prognosis in LGG (Figure 5J), and, Neutrophil infiltration and MYL9 expression was markedly correlated with prognosis in MESO (Figure 5K). This suggested that MYL9 played a strong role in regulating immune cell infiltration, with a particularly strong effect on CD8^+^T cell infiltration in BLCA, on B cell, CD8^+^T cell, CD4^+^T cell, macrophage, neutrophil, DC infiltration in LGG, and on Neutrophil infiltration in MESO.

### 3.7 The enrichment analysis of MYL9‐related gene

**Figure 6:**
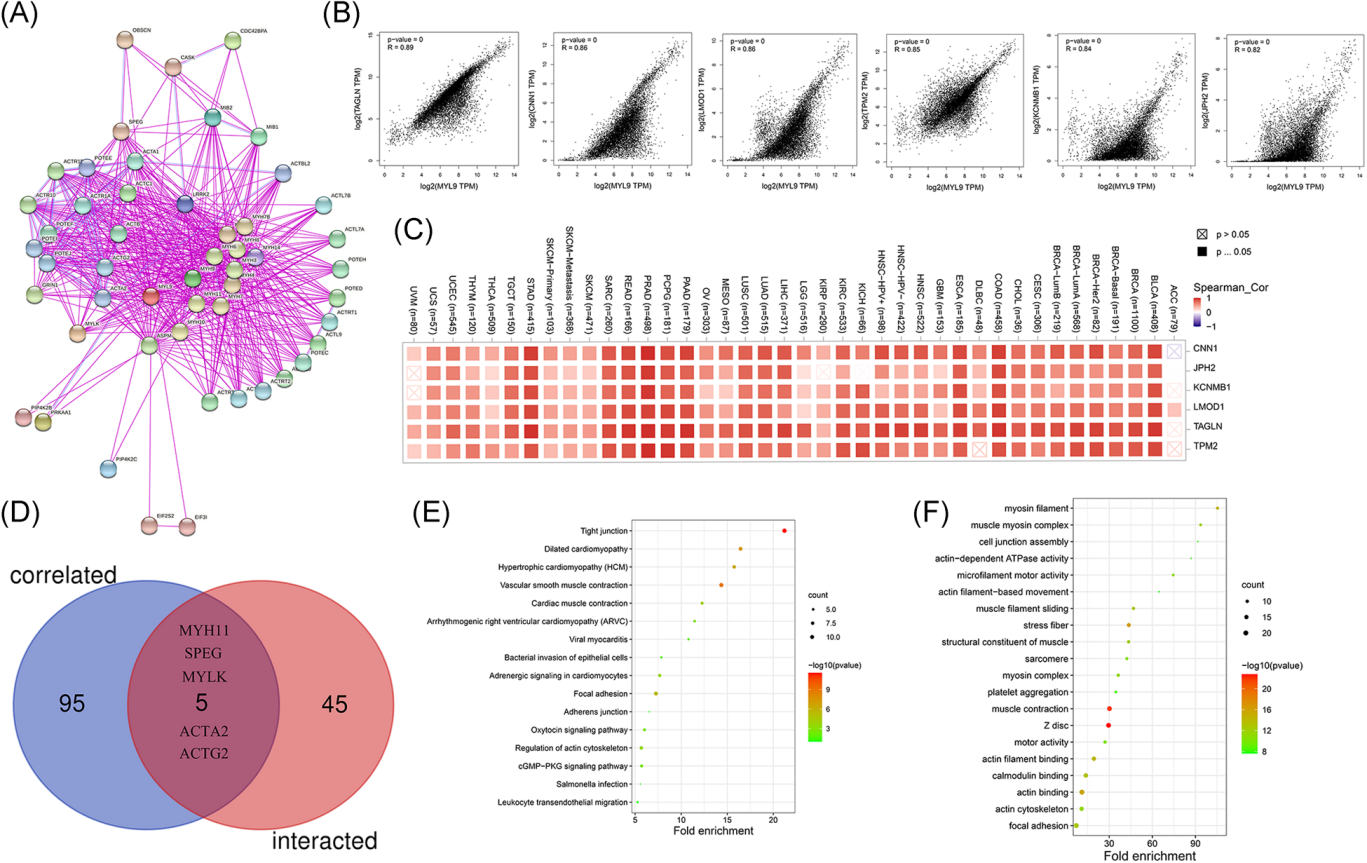
The enrichment analysis of the myosin light chain 9 (MYL9)‐related gene. (A) The available experimentally determined MYL9‐binding proteins were obtained by using the STRING tool. (B) The correlation analysis between the top 6 gene (TAGLN, CNN1, LMOD1, TPM2, KCNMB1, and JPH2) of those correlated with MYL9 expression and MYL9 via using GEPIA2. (C) The correlation analysis between the top 6 gene (TAGLN, CNN1, LMOD1, TPM2, KCNMB1, and JPH2) of those correlated with MYL9 expression and MYL9 via using TIMER 2.0. (D) Venn diagram showed an intersection analysis of the MYL9 binding and correlated genes. KEGG pathway analysis (E) and GO analysis (F) were performed based on the MYL9 binding and interacted genes

To further study the molecular mechanism of MYL9 in tumorigenesis, we attempted to screen out targeted MYL9 binding proteins and the related genes of MYL9 expression for pathway enrichment analysis. As shown in Figure 6A, through employing the STRING tool, we obtained a total of 50 MYL9‐binding proteins, and showed the interaction network of these proteins. In addition, we used the GEPIA2 tool to combine all tumor expression data of TCGA and obtained the top 100 genes that correlated with MYL9 expression. Then, the top 6 gene of those correlated with MYL9 expression were further analyzed by the GEPIA2 tool for correlation, and the results were shown by scatter diagram (Figure 6B), including TAGLN (transgelin), CNN1 (calponin 1), LMOD1 (leiomodin 1), TPM2 (tropomyosin 2), KCNMB1 (potassium calcium‐activated channel subfamily M regulatory beta subunit 1), and JPH2 (junctophilin 2). The corresponding heatmap data also showed a positive correlation between MYL9 and the above six genes in most detailed cancer types (Figure 6C). Using the Venn diagrams, an intersection analysis of the above two groups showed five common members, namely, MYH11 (myosin heavy chain 11), SPEG (striated muscle enriched protein kinase), MYLK (myosin light chain kinase), ACTA2 (actin alpha 2), and ACTG2 (actin gamma 2) (Figure 6D). To further study the pathogenesis of MYL9 gene in tumors, we combined the two datasets to perform KEGG and GO enrichment analyses. As shown in Figure 6E, The bubble diagram of KEGG data suggested that “tight junction,” “vascular smooth muscle contraction,” “focal adhesion,” and “dilated cardiomyopathy” might be involved in the effect of MYL9 on tumor pathogenesis, and “Oxytocin and cGMP‐PKG signaling pathway” might be involved in the majority signaling pathway for tumorigenesis mechanism. In the GO data set, we found that “muscle contraction,” “myosin filament,” “focal adhesion,” and “calmodulin binding” might be involved in the effect of MYL9 on tumor pathogenesis (Figure 6F). The combined information of the two databases manifested that the effects of MYL9 gene on oncogenesis and progression mainly involved muscle contraction and focal adhesion, which will provide a direction for clinical diagnosis and treatment of cancer patients.

## 4 DISCUSSION

It has been reported that the myosin family was subdivided into two groups: Class I (small, mostly monomeric motors) and Class II (dimeric myosins similar to skeletal muscle myosin; this class includes both muscle and nonmuscle myosins).^13^ Class II myosin, also known as traditional myosin, is a hexameric molecule composed of two heavy chains and two pairs of light chains, including the essential light chain and the regulatory light chain.^14^ Myosin II activity is mediated primarily by posttranslational phosphorylation of MYL9 (also known as MLC2, MRLC1, or MLC‐2C) and by the opposite activity of MLC kinase and an MLC phosphatase.^15^

MYL9 played an important role not only in oncogenesis but also could be used as a sensitive biomarker for tumor diagnosis. For example, in colon cancer, MYL9 expression was downregulated in tumor tissues compared with normal tissues and the area under the curve (AUC) of MYL9 in diagnosis for colon cancer was 0.826, indicating statistical significance.^16^ A study of MYL9 in nonsmall cell lung cancer (NSCLC) showed that the expression levels of and MYL9 were significantly lower in cancer tissue than those in the paraneoplastic and normal tissues, suggesting that low MYLK and MYL9 expressions might be associated with the development of NSCLC.^17^ Studies exhibited that MYL9 was involved in regulating breast cancer invasion,^18^ and MYL9 depletion did not influence cell cycle progression or induce cell death in MDA‐MB‐231 cells but obviously reduced invasiveness.^19^ In the present study, we found that the expression levels of the MYL9 gene were also downregulated in COAD compared with the corresponding normal tissues, the OS (*p* = .0061) and DFS (*p* = .019) of the low MYL9 expression group were longer than the high MYL9 expression group, which were in accordance the result about the DFS of colon cancer patients in PrognoScan database and suggested that MYL9 could be used as a biomarker for COAD diagnosis and therapy. The OS of low MYL9 gene expression group in LGG (*p* = .0015) and MESO (*p* = .0054) was obviously longer than that in the MYL9 high expression group, while it became shorter in ACC (*p* = .025). Interestingly, the DFS of low MYL9 gene expression group in THYM (*p* = .013) was longer than the high MYL9 gene expression group. Therefore, the value of MYL9 for diverse tumor diagnosis and therapy was different, which deserved further investigation. The results from Prognoscan and Kaplan–Meier plotter databases showed that MYL9 expression correlated significantly with prognosis in lung cancer, breast cancer, colorectal cancer, brain glioma, prostate cancer, especially ovarian cancer and gastric cancer. We further used the Kaplan–Meier plotter tool to study the relationship between MYL9 expression and different clinicopathological factors in gastric and ovarian cancer, finding that MYL9 expression may affect the prognosis of gastric cancer patients by affecting lymph node metastasis.

It is well known that tumor infiltrating immune cells, as an important part of the tumor microenvironment, are closely related to tumor genesis, development, or metastasis.^20^, ^21^ All stromal cells are clustered in the tumor microenvironment, while cancer‐related fibroblasts (CAFs) are the most abundant and play a key role in cancer progression.^22^ In this study, we explored the correlation between MYL9 gene expression and CAFs in all types of cancer in the TCGA dataset, finding that there is a significant positive correlation between the two in all kinds of tumor except SARC. In addition, we further employed the “Outcome” model of TIMER2.0 tool to analyze the effects of CAFs, age, gender, race, purity, and MYL9 on prognosis correlation by setting up multivariable Cox Proportional Hazard Models. Results showed that the level of MYL9 expression and the degree of CAFs infiltration affected the CS of cancer patients, and they played different or adverse roles in different tumors. Recent studies showed that the CD69‐Myl9 system in immune responses could be used as a new therapeutic target for intractable inflammatory disorders and tumors.^23^ In this study, we also investigated the effects of the immune infiltrating of B cell, CD8^+^T cell, CD4^+^T cell, macrophage, neutrophil, DC, and the expression of MYL9 on CS in different cancers, and results indicated that MYL9 played a strong role in regulating immune cell infiltration, with a particularly strong effect on CD8^+^T cell infiltration in BLCA, on B cell, CD8^+^T cell, CD4^+^T cell, macrophage, neutrophil, DC infiltration in LGG, and on neutrophil infiltration in MESO. Furthermore, we found that MYL9 expression correlated with the markers of monocyte, TAM, M1 and M2 macrophage, Tregs, and exhausted T cells, indicating that MYL9 may be capable of regulating the polarization of macrophages and may play a role in immune escape in STAD and COAD. Therefore, our data manifested that the possibility of MYL9 combined with immune infiltration cells could be used as a new therapeutic target for patients with tumors.

Our previous data manifested that the MYL9 gene was associated with the prognosis in certain tumors. Subsequently, we integrated information on MYL9 binding components and genes associated with MYL9 expression in all tumors and performed a series of enrichment analyses to identify the potential role of muscle contraction and local adhesion in cancer etiology or pathogenesis. However, we just used public databases of Oncomine, TCGA, GEO, PrognoScan, and, Kaplan–Meier plotter datasets to illustrate the effects of the MYL9 gene on different tumors. When encountering problems in a particular tumor in clinical practice, we still need more evidence in cell and animal levels for detailed mechanism studies.

## 5 CONCLUSION

In conclusion, our first pan‐cancer analysis of MYL9 demonstrated statistical correlations of MYL9 expression with clinical prognosis, immune infiltrating across multiple cancers, which aids in understanding the role of MYL9 in tumorigenesis from the perspective of clinical cancer samples. MYL9 can serve as a prognostic signature in pan‐cancer and is associated with immune infiltrating.

## AUTHOR CONTRIBUTIONS

*Data curation, formal analysis, investigation, methodology, software, visualization, and writing—original draft*: Minghe Lv. *Project administration*: Xue Chen. *Supervision*: Xue Chen. *Validation*: Lumeng Luo. *Writing—review and editing*: Minghe Lv and Xue Chen.

## Supporting information

Supporting information.

## Data Availability

The datasets generated and analyzed during the current study are available in TCGA, GEO, Oncomine, GTEx, GEPIA 2, TIMER, TIMER2.0, PrognoScan, Kaplan–Meier Plotter, CPTAC, STRING, DAVID, KEGG, and GO datasets.
