# Supplementary material for: The landscape of prognostic and immunological role of myosin light chain 9 (MYL9) in human tumors
Source: Immun Inflamm Dis. 2021 Nov 2;10(2):241–54. doi: 10.1002/iid3.557 (PMC8767521; doi:10.1002/iid3.557)
Supplement: Supplementary file 1 — Supporting information. [file IID3-10-241-s001.pdf]

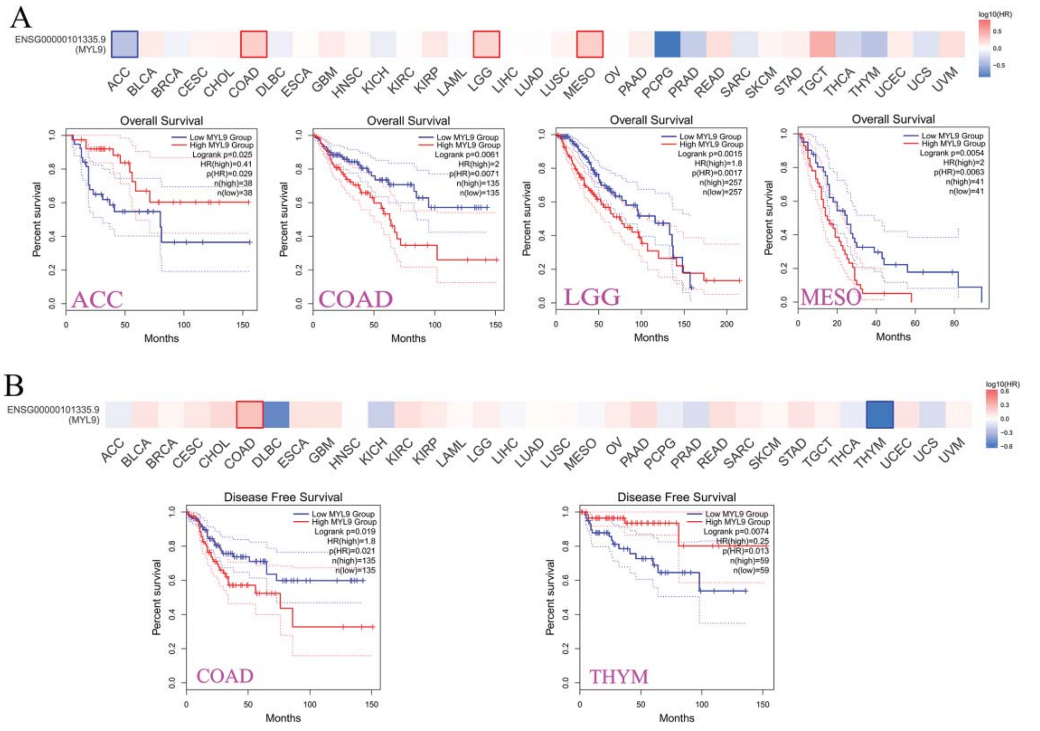

Figure S1 The prognosis analysis of MYL9 gene expression in TCGA dataset for different tumors.

view Only

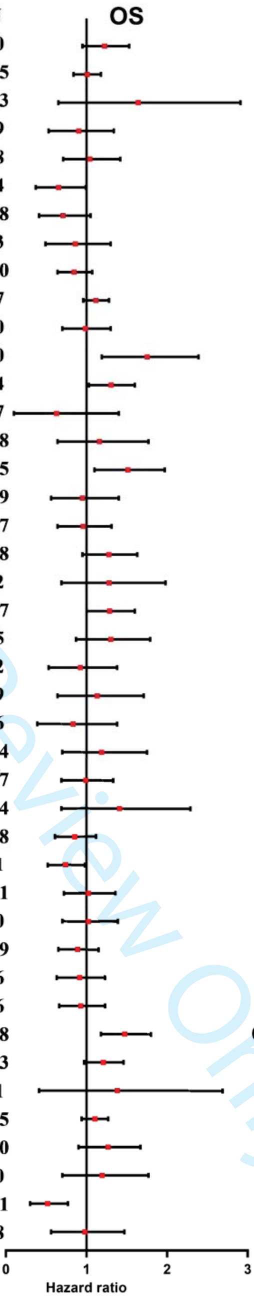

Cancer Type	Dataset	N	P-Value	Hazard ratio
Bladder cancer	GSE5287	30	0.130237	1.20 [0.95 - 1.53]
Bladder cancer	GSE13507	165	0.97241	1.00 [0.84 - 1.18]
Blood cancer	GSE12417-GPL96	163	0.406895	1.37 [0.65 - 2.91]
Blood cancer	GSE12417-GPL570	79	0.469806	0.84 [0.53 - 1.34]
Blood cancer	GSE5122	58	0.98263	1.00 [0.71 - 1.42]
Blood cancer	GSE8970	34	0.0474035	0.60 [0.37 - 0.99]
Blood cancer	GSE4475	158	0.0760959	0.66 [0.41 - 1.05]
Blood cancer	E-TABM-346	53	0.36531	0.80 [0.49 - 1.30]
Blood cancer	GSE16131-GPL96	180	0.15488	0.83 [0.64 - 1.07]
Brain cancer	GSE4271-GPL96	77	0.172076	1.11 [0.96 - 1.28]
Brain cancer	GSE7696	70	0.76797	0.96 [0.70 - 1.30]
Brain cancer	MGH-glioma	50	0.00365369	1.68 [1.19 - 2.39]
Brain cancer	GSE4412-GPL96	74	0.0233779	1.29 [1.03 - 1.60]
Brain cancer	GSE16581	67	0.147951	0.38 [0.10 - 1.40]
Breast cancer	GSE3143	158	0.804896	1.07 [0.64 - 1.77]
Breast cancer	GSE9893	155	0.00884911	1.47 [1.10 - 1.97]
Breast cancer	GSE1456-GPL96	159	0.608859	0.89 [0.56 - 1.40]
Breast cancer	E-TABM-158	117	0.638817	0.92 [0.64 - 1.31]
Breast cancer	GSE7390	198	0.106881	1.25 [0.95 - 1.63]
Colorectal cancer	GSE12945	62	0.554726	1.17 [0.69 - 1.98]
Colorectal cancer	GSE17536	177	0.0507732	1.26 [1.00 - 1.60]
Colorectal cancer	GSE17537	55	0.238269	1.24 [0.87 - 1.79]
Lung cancer	jacob-00182-CANDF	82	0.524145	0.86 [0.53 - 1.38]
Lung cancer	jacob-00182-HLM	79	0.860687	1.05 [0.64 - 1.71]
Lung cancer	MICHIGAN-LC	86	0.331989	0.73 [0.39 - 1.38]
Lung cancer	jacob-00182-MSK	104	0.67147	1.11 [0.70 - 1.75]
Lung cancer	GSE13213	117	0.809186	0.96 [0.69 - 1.33]
Lung cancer	GSE31210	204	0.459679	1.25 [0.69 - 2.29]
Lung cancer	jacob-00182-UM	178	0.226014	0.83 [0.61 - 1.12]
Lung cancer	GSE11117	41	0.0362181	0.72 [0.52 - 0.98]
Lung cancer	GSE3141	111	0.942791	0.99 [0.72 - 1.36]
Lung cancer	GSE14814	90	0.927729	0.98 [0.70 - 1.39]
Lung cancer	GSE4573	129	0.315487	0.87 [0.65 - 1.15]
Lung cancer	GSE17710	56	0.457803	0.88 [0.63 - 1.23]
Lung cancer	GSE17710	56	0.513128	0.90 [0.66 - 1.23]
Ovarian cancer	GSE9891	278	0.000520198	1.45 [1.18 - 1.80]
Ovarian cancer	DUKE-OC	133	0.105088	1.19 [0.97 - 1.46]
Ovarian cancer	GSE8841	81	0.923892	1.05 [0.41 - 2.69]
Ovarian cancer	GSE26712	185	0.229562	1.10 [0.94 - 1.27]
Ovarian cancer	GSE17260	110	0.197443	1.23 [0.90 - 1.67]
Ovarian cancer	GSE14764	80	0.645762	1.11 [0.70 - 1.77]
Prostate cancer	GSE16560	281	0.0021093	0.48 [0.30 - 0.77]
Skin cancer	GSE19234	38	0.69089	0.91 [0.56 - 1.47]

For Review Only

Figure S2 Relation between MYL9 expression and patient prognosis of different cancer (Overall Survival) in Prognoscan database.

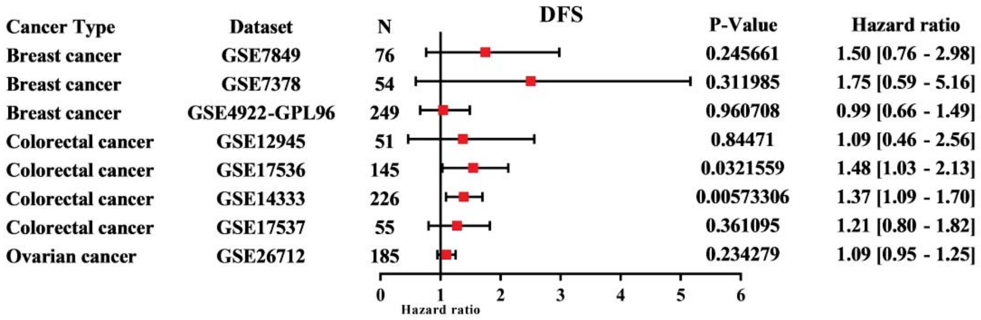

Figure S3 Relation between MYL9 expression and patient prognosis of different cancer (Disease Free Survival) in Prognoscan database.

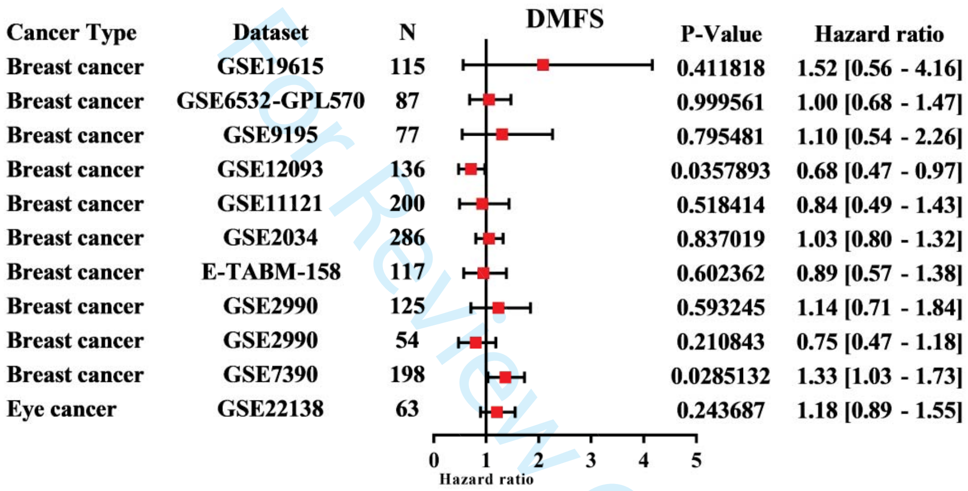

Figure S4 Relation between MYL9 expression and patient prognosis of different cancer (Distant Metastasis Free Survival) in Prognoscan database.

For Review Only

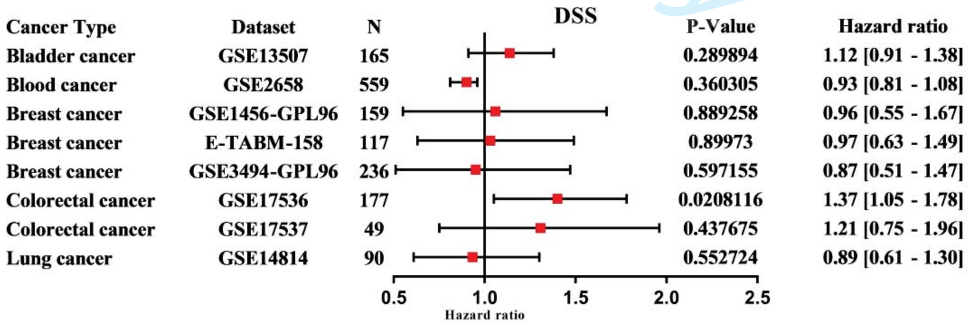

Figure S5 Relation between MYL9 expression and patient prognosis of different cancer (Disease Specific Survival) in Prognoscan database.

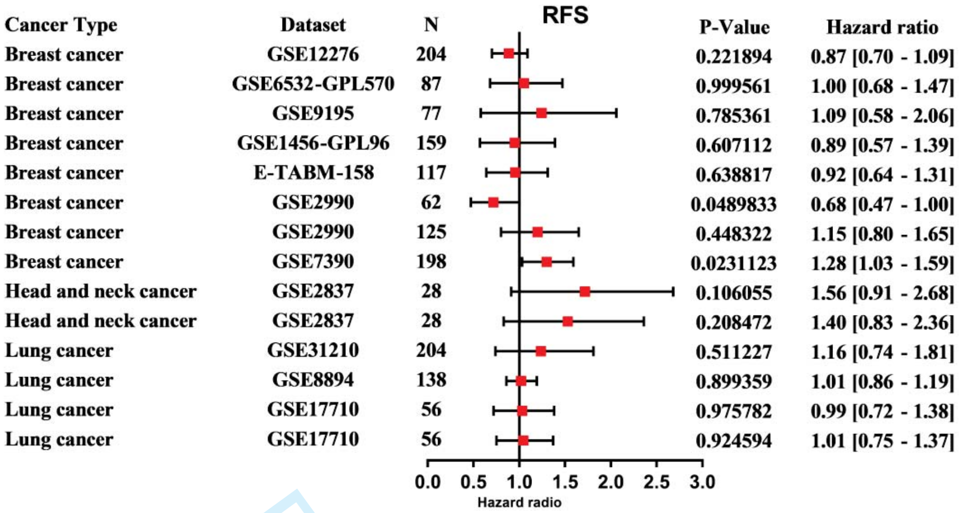

Figure S6 Relation between MYL9 expression and patient prognosis of different cancer (Relapse Free Survival) in Prognoscan database.

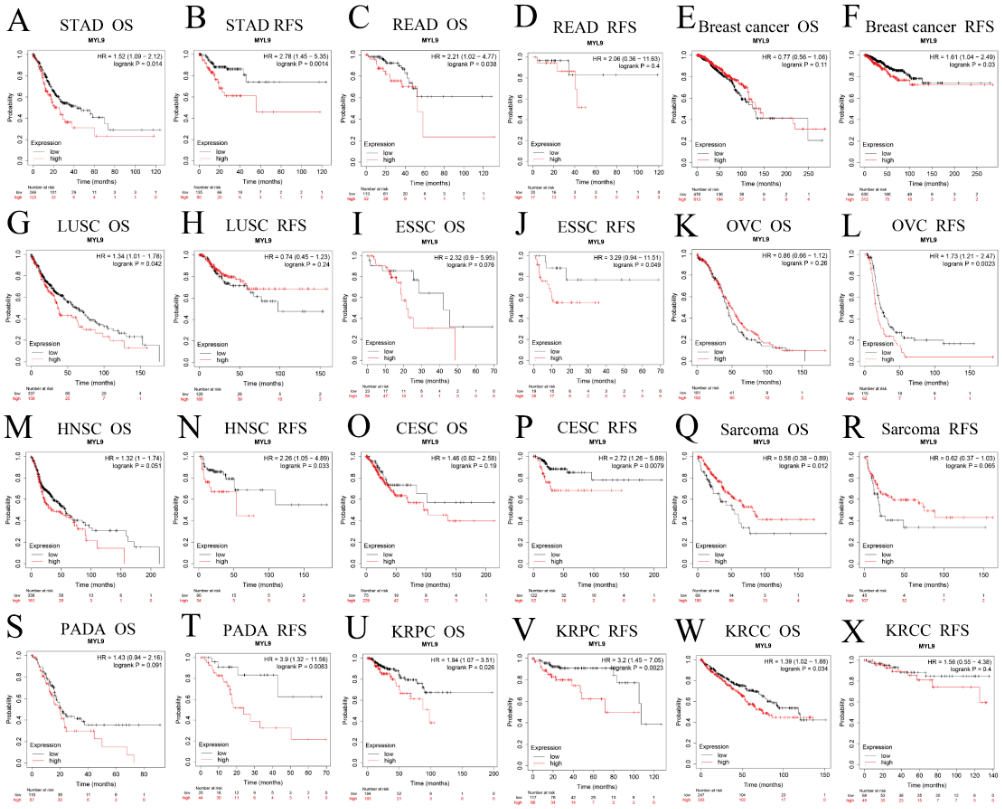

Figure S7 Survival analysis data of MYL9 mRNA expression in Kaplan-Meier RNA-seq dataset

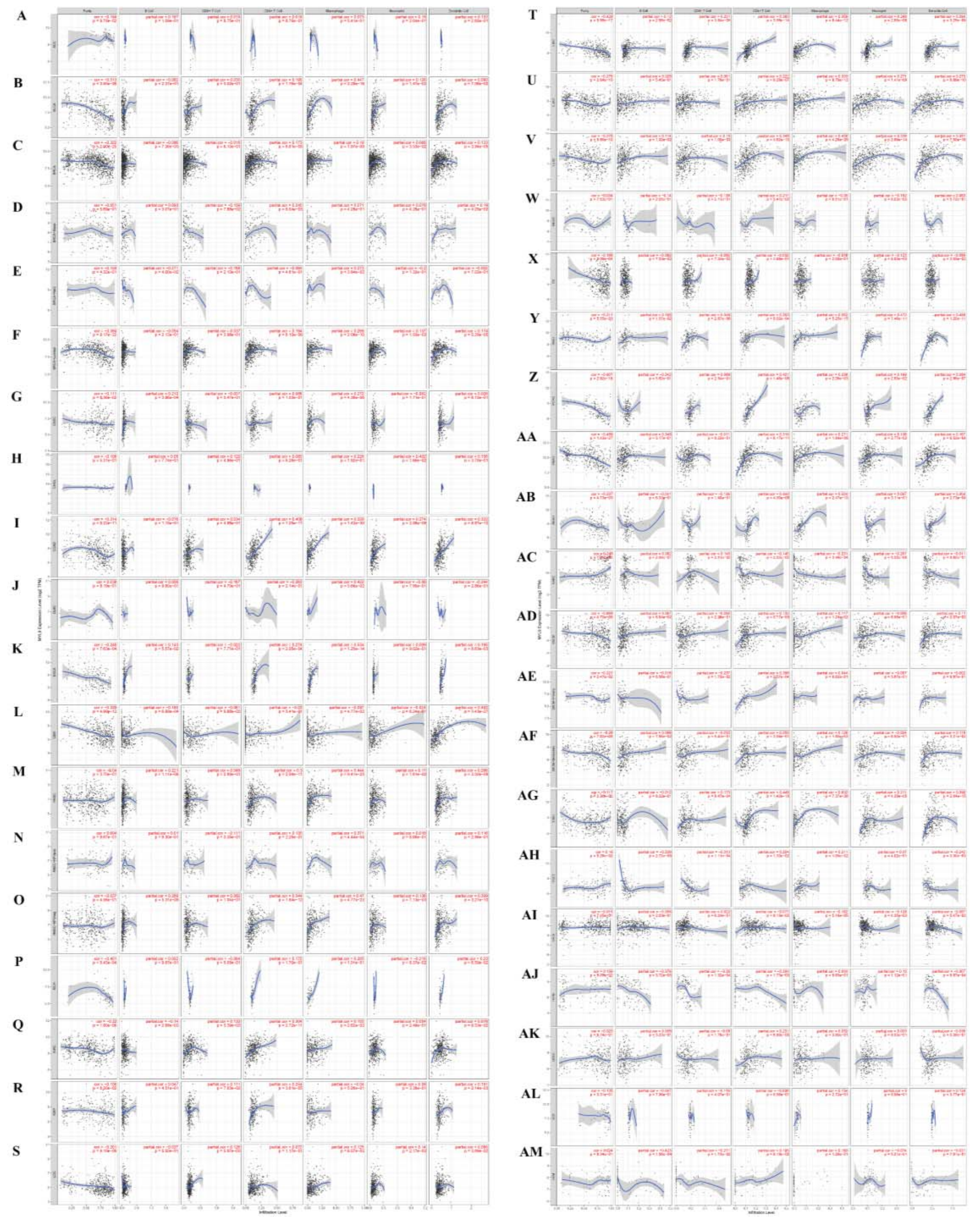

Figure S8 Correlation of MYL9 expression with immune infiltration levels in TIMER.

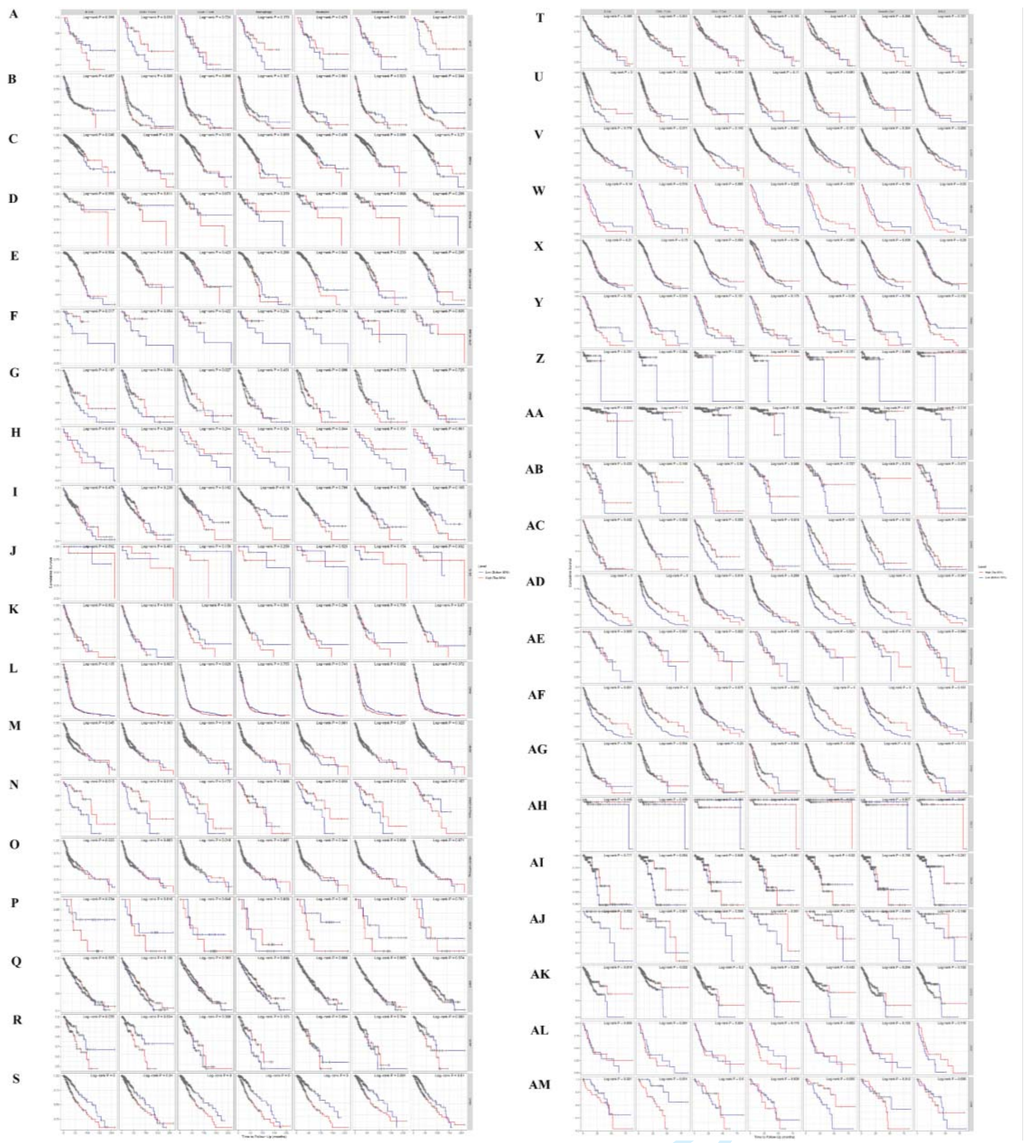

Figure S9 Kaplan-Meier plots of immune infiltration and MYL9 expression levels in TIMER.
